# *Theobroma cacao* L. cultivar CCN 51: a comprehensive review on origin, genetics, sensory properties, production dynamics, and physiological aspects

**DOI:** 10.7717/peerj.12676

**Published:** 2022-01-05

**Authors:** Ramon E. Jaimez, Luigy Barragan, Miguel Fernández-Niño, Ludger A. Wessjohann, George Cedeño-Garcia, Ignacio Sotomayor Cantos, Francisco Arteaga

**Affiliations:** 1 Facultad de Ingeniería Agronómica, Universidad Técnica de Manabí, Manabí, Ecuador; 2 Mars La Chola MLCH C.L, Guayas, Ecuador; 3 Department of Bioorganic Chemistry, Leibniz-Institute of Plant Biochemistry, Halle (Saale), Germany; 4 Estación Experimental Tropical Pichilingue, Instituto Nacional de Investigaciones Agropecuarias (INIAP), Los Ríos, Ecuador

**Keywords:** Theobroma cacao, Ecuadorian cacao, Cocoa, CCN 51, Cacao ecophysiology, Chocolate quality

## Abstract

Many decades of improvement in cacao have aided to obtain cultivars with characteristics of tolerance to diseases, adaptability to different edaphoclimatic conditions, and higher yields. In Ecuador, as a result of several breeding programs, the clone CCN 51 was obtained, which gradually expanded through the cacao-production regions of Ecuador, Colombia, Brazil and Peru. Recognized for its high yield and adaptability to different regions and environments, it has become one of the most popular clones for breeding programs and cultivation around the world. This review aims to summarize the current evidence on the origin, genetics, morphological, volatile compounds, and organoleptic characteristics of this clone. Physiological evidence, production dynamics, and floral biology are also included to explain the high yield of CCN 51. Thus, characteristics such as osmotic adjustment, long pollen longevity, and fruit formation are further discussed and associated with high production at the end of the dry period. Finally, the impact of this popular clone on the current and future cacao industry will be discussed highlighting the major challenges for flavor enhancement and its relevance as a platform for the identification of novel genetic markers for cultivar improvement in breeding programs.

## Introduction

The recent archaeological evidence shows the use of cacao (*Theobroma cacao* L.) 5,300 years ago in Santa Ana-La Florida in the Zamora-Chinchipe province in the South-East of Ecuador, the upper part of the Amazon (Zarrillo et al., 2018). Cacao was probably already cultivated by the year 1600 and the largest Ecuadorian cacao productions were plantations located in the tributaries upstream of Guayaquil city. At that time, Ecuadorian cacao was known as ‘*Cacao de Arriba’* (name attributed to the high-quality cacao produced in the Ecuadorian district “*Arriba”* (currently known as Los Rios Province)). Higher production and quality of cacao in this province compared with other production provinces (*e.g.*, Machala, Manabí, and Balao) were attributed to a deep alluvial soil, better drainage due to its higher altitude above sea level, a low prevalence of strong winds, and high rainfall (Maiguashca, 2014). In 1890, Ecuadorian cacao exports increased to 12000 t compared to the 6000 t that was produced in 1850 (Maiguashca, 2014). Over time, Ecuador began to have a large number of cacao cultivars as a result of the hybridization of Ecuadorian “Nacional” cacaos with Trinidadian, Venezuelan, and Amazonian cultivars, introduced in the 1940s (Arosemena, 1991). Since that time, several international and national cacao collections were established, which provided an opportunity for Ecuador to carry out breeding programs to obtain clones that were evaluated in various locations. In these programs, Nacional-type cultivars were first selected based on their yield, which exceeded the national averages of that time and delivered to the producers by the Instituto Nacional de Investigaciones Agropecuarias (INIAP) at the end of the 1950s. The breeding programs continued with the idea to achieve Nacional cacaos with higher yields, and for the first time in the 70s, INIAP released Nacional-type clones to producers. In this period, there were also breeding trials carried out by personal initiatives and private companies. From these the clone CCN 51 emerged, a clone of high yield, interestingly unrelated to Nacional cacao. This clone showed its great adaptability to a variety of climates and today it is cultivated in all cacao regions of Ecuador (Amores et al., 2011; Sánchez-Mora et al., 2015), in some regions of Colombia (Sánchez et al., 2007; Quintana Fuentes et al., 2015a), Peru (Tuesta Hidalgo et al., 2014; Hernández Calderón & Gonzáles Hidalgo, 2016), Brazil (Reges et al., 2021) and some Central American countries such as Guatemala. CCN 51 has physiological, floral biology, and production characteristics that give it greater productivity and adaptation to different environments varying from areas characterized by high rainfall to others with dry climates. Little research has been performed to dissect the physiological characteristics and production dynamics that give CCN 51 its high performance in different edaphoclimatic conditions. The objective of this review is therefore to discuss the evidence that until now exists in gas exchange, floral biology, water relations, and production dynamics, to explain how they interrelate and contribute to the high yields of CCN 51. Firstly, the origin, genetic and organoleptic characteristics of this popular clone are summarized. Then, the behavior of physiological traits (*i.e.,* CO2 assimilation rates (A), water relations, chlorophyll (Chla) parameters, and osmotic adjustment) in periods of dry and rain in different environments are shown. In addition, the production dynamics and its relationship with floral biology are also analyzed to show its implications in greater productions. Finally, the relevance of this clone in the current and future cacao industry is discussed based on the reviewed data. This manuscript represents the first comprehensive review for this widespread popular clone, and it will constitute a valuable tool for future physiological, breeding, genetic, biochemical, and sensory studies.

## Rationale for the Current Review

As mentioned before, the popularity of CCN 51 has been increased worldwide (especially in Latin America) due to its high productivity, resistance to diseases, and adaptability to a large number of ecogeographical regions and environments. Consequently, the number of hectares planted with this popular clone has increased, thus contributing to the regional rise in cacao production. At the same time, small producers have placed their expectations on this clone in hope of achieving a significant reactivation of their farms. This manuscript represents the first comprehensive review for this widespread popular clone. Here, we summarize the current evidence on the origin, genetics, morphological and organoleptic characteristics of CCN 51. Its relevance in the current and future cacao industry is discussed based on the reviewed data. Moreover, we also highlighted the major challenges for its flavor enhancement and its application as a platform for cultivar improvement in breeding programs.

This article is intended for cacao researchers around the world and, it will be also beneficial to local cacao producers and cacao companies specially located in Latin America.

## Search Methodology

Literature was reviewed using six electronic databases (*i.e.,* PubMed, Google Scholar, EBSCO, ScienceDirect, Wiley, and SciELO). English and Spanish language articles (published in peer-reviewed journals between earliest record and August 1, 2021). Literature was searched using (“CCN 51” OR “Theobroma cacao CCN 51”). AND (“genetics” OR “origin” OR “sensory”, “flavor” OR “QTL mapping” OR “physiology” OR “production” OR “floral biology” OR “yield” OR “osmotic adjustment” OR “fruit formation”OR “gas exchange”OR “pollen longevity” OR “cacao industry” OR “fine-flavour” OR “breeding”) as search terms in the title and abstract. Duplicated publications were manually removed by REJ and MFN, thus obtaining a total of 82 peer-reviewed selected publications. This review includes four main sections: first, a compressive review of CCN 51 relevance, origin, and genetics, second, a review of its sensory profile and flavor chemistry, third, a review on its physiological characteristics and finally, a conclusion section on current challenges and perspectives for CCN 51 utilization.

## The Relevance of CCN 51 for Cacao Production in Latin America

**Figure 1:**
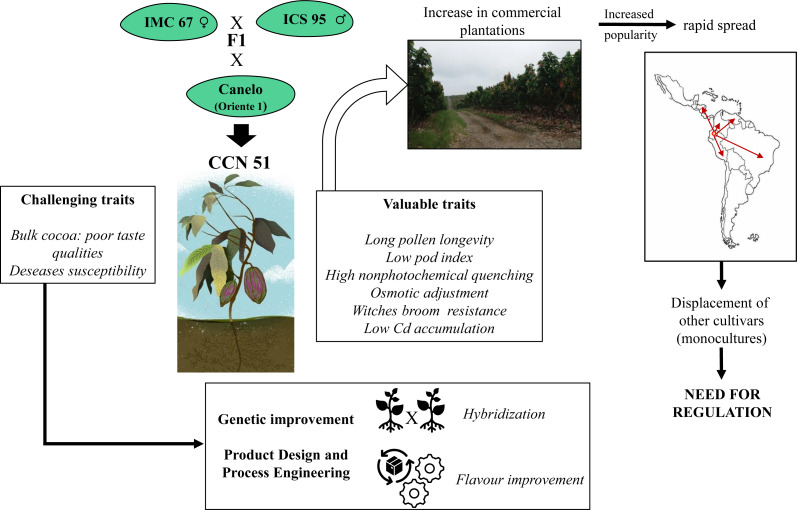
Origin, distribution, and relevance of CCN 51 for cacao production in Latin America. CCN 51 was obtained from the crosses (IMC-67 X ICS-95) x “Canelo” (Oriente 1) in the Naranjal region (Guayas Province, Ecuador). The rapid spread of this clone in Latin America has been mainly associated with its valuable traits. Additional research is still required to improve CCN 51 challenging traits and regulate its utilization.

Possibly the creator of CCN 51, Homero Castro Zurita, did not imagine that this clone would be so productive and adaptable to a wide variety of places (Fig. 1), as has been extensively reported (Boza et al., 2014; Díaz-Valderrama, Leiva-Espinoza & Catherine Aime, 2020; Fernandes et al., 2020; Reges et al., 2021). As shown in Fig. 1, he obtained this clone from the crosses (IMC-67 X ICS-95) x “Canelo” (Oriente 1) in his Theobroma farm in the Naranjal region (Guayas Province, Ecuador) in response to a devastating incidence of witches’ broom disease in Ecuador by 1960 (Castro, 1981). The “Sofia” farm, located in the Naranjal region, was the first farm to be reported with CCN 51 plantations in 1965 (Crespo del Campo & Crespo Andia (1997)). Its commercial plantations began in Ecuador in 1985, and thirty years later, by 2015, the Association of cacao exporters of Ecuador (ANECACAO) reported an estimated cacao production of 260000 t, of which 30% corresponded to CCN 51, that is, 120000 t. In 2018, the National Institute of Statistics and Censuses of Ecuador (INEC) reported 573000 hectares of cacao, of which 70% corresponded to CCN 51. It is also worth noting that 54% of Peruvian production, which is 84700 t, comes from CCN 51 (Hernández Calderón & Gonzáles Hidalgo, 2016) and the popularity of this clone is also increasing in other countries of the region (Fig. 1) including Colombia, Panama, Guatemala, and Brazil (Sanchez, Zambrano & Iglesias, 2019). The rapid spread of this clone in Latin America has been associated with its valuable traits as described in Fig. 1. One of the most important traits of CCN 51 is its remarkable productivity, with estimated yields of 1300-1800 kg/ha, which on average is higher than for other economically relevant clones in the region, as reported for Ecuador (Ramlachan et al., 2009; Boza et al., 2014; Sánchez-Mora et al., 2015; Jaimez et al., 2018), Colombia (Puentes-Páramo, Menjivar-Flores & Aranzazu-Hernández, 2014) and Brazil (Reges et al., 2021)

## Origin and Genetics

As mentioned before, CCN 51 is traditionally recognized as a clone originated from the triple cross of (IMC-67 X ICS-95) ×“Canelo” (Oriente 1). ICS-95 (Imperial college selection) is a Trinitario clone with parental contributions from lower Amazon Forastero and Criollo (Lanaud, Motamayor & Risterucci, 2001), whereas IMC-67 is an upper Amazon Forastero collected from Peru (Lanaud, Motamayor & Risterucci, 2001; Bekele & Phillips-Mora, 2019). In 2014, Boza and co-workers reported novel molecular evidence on the origin of this clone by multilocus genotyping and studying its gene structure (Boza et al., 2014). In their work, they confirmed the clones IMC 67 and ICS 95 as parentals for CCN 51. Nuclear, mitochondrial, and chloroplast DNA analysis revealed IMC 67 (*i.e.,* the maternal allele donor with a significant contribution to the global genetic structure) as a principal genetic origin followed by ICS 95 (a cultivar that originated from the hybridization of Criollo and Amelonado) and finally some alleles related to the Iquitos, Criollo, and Amelonado genetic groups. These groups belong to the new classification proposed by Motamayor et al. (2008) that reflects the existence of genetic diversity which is very different from that previously proposed with only two segregated groups (Criollo and Forasteros) (Motamayor et al., 2008). Interestingly, the Oriente 1 cultivar (collected by Castro in the Ecuadorian Amazon) (Castro, 1981) was not found in germplasm collections, thus the authors did not find any genetic information that can be related to this clone (Boza et al., 2014).

**Figure 2:**
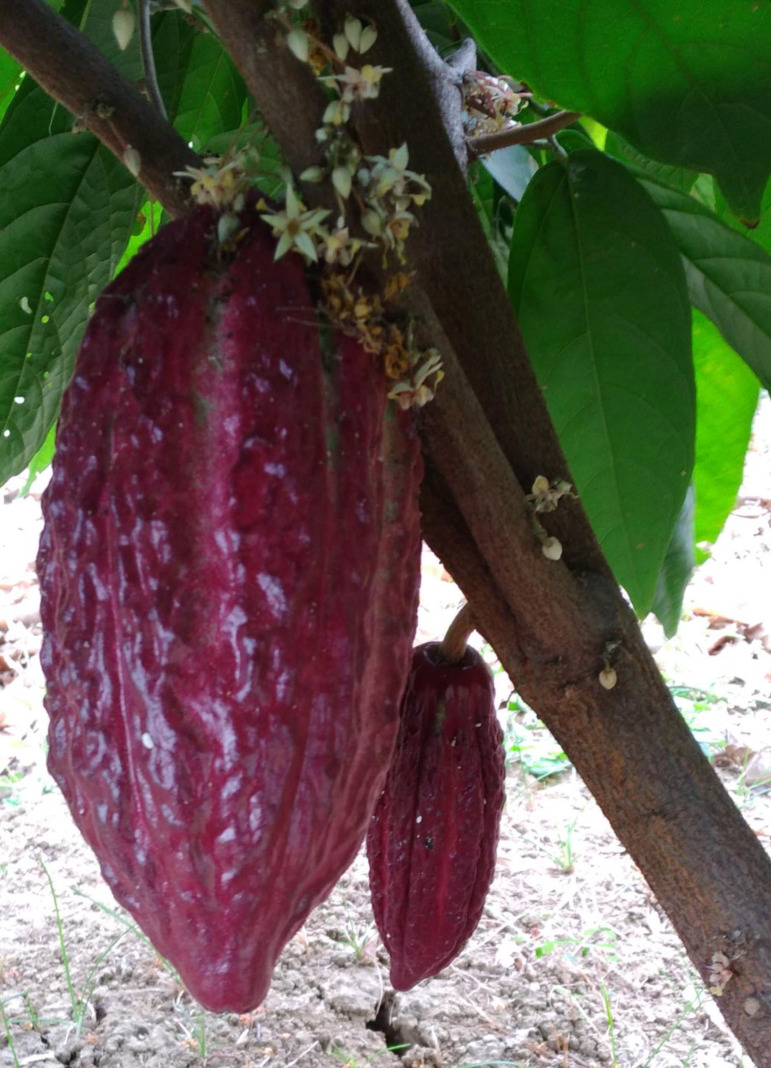
CCN 51 oblong-shaped fruits with an obtuse apex. Pictures of plants growing at the Universidad Técnica de Manabí, Ecuador.

The work from Boza et al. (2014) also highlighted the wide differences between CCN 51 and the Nacional fine-flavor cultivars. Unlike Nacional cultivars, CCN 51 has high heterozygosity (80%) and only 1.1% of its analyzed alleles were associated with the Nacional ones (Boza et al., 2014). CCN 51 is highly appreciated for its high tolerance to diseases and its productivity, however, it is currently considered as a “bulk” type cacao lacking the valuable fine-flavor profile of Nacional types as will be discussed below (Quintana Fuentes et al., 2015b; (Perea et al., 2017; Rottiers et al., 2019). Unfortunately, intermixing CCN 51 and fine-flavor cultivars has become a frequent practice by some local farmers in Ecuador, which is currently affecting its value as a premium commodity (Motamayor et al., 2013; Nader, Brendel & Schubbert, 2016). Consequently, several alternatives have been developed to identify adulteration of Nacional beans with CCN 51. A popular method relies on the phenotypic evaluation of CCN 51 traits (*e.g.*, yield, beans weight, pod color, and flavor). Thus, CCN 51 cultivar is distinguished by its oblong-shaped fruits, with an obtuse apex, red when immature, and orange when they reach maturity (Fig. 2). It has purple seeds with an average length of 25 mm and a diameter of 14 mm (Perea et al., 2017). This clone is also recognized by its heavier beans as compared to other clones, which has been recently reported as an attribute in clones with Criollo ancestry (Doaré, Ribeyre & Cilas, 2020). Nevertheless, phenotypic evaluation is an expensive and vague method, even when a quick method using computational images has been also developed to differentiate CCN 51 and Nacional beans in mixtures (Jimenez et al., 2018a). Consequently, several molecular-based approaches have been developed recently for CCN 51 identification (Herrmann et al., 2014; Vargas Jentzsch et al., 2016; Scharf, Lang & Fischer, 2020; Stagnati et al., 2020). These methods include DNA fingerprinting of liquor using Simple Sequence Repeats (SSR) profiling (Stagnati et al., 2020), Single Nucleotide Polymorphisms (SNPs) profiling of chloroplast DNA (Herrmann et al., 2014), Raman Spectroscopy of cacao beans (Vargas Jentzsch et al., 2016), and even CRISPR/Cas9-based *in vitro* approach targeting specific SNPs in CCN 51 DNA (Scharf, Lang & Fischer, 2020).

The characterization of several DNA sequences for CCN 51 traceability has improved the knowledge regarding SNPs that can be used for mapping experiments aimed to identify genetic determinants associated with the higher tolerance to diseases and productivity in other cultivars using the CCN 51 as reference parental. Thus, in the last six years, several Quantitative Trait Loci (QTL) mapping experiments have been performed using CCN 51 as one of the parental, which allowed the identification of genes in other cultivars responsible for tolerance to Witches’ broom disease (Royaert et al., 2016; Santana et al., 2018), black pod disease (Barreto et al., 2015; Barreto et al., 2018; Villamizar-Gallardo, Osma & Ortíz-Rodriguez, 2019), Ceratocystis wilt Disease (Fernandes et al., 2018), fat content and fatty acid composition (Mustiga et al., 2019) and recently the genetic basis for exceptional yield (Fernandes et al., 2020). These kinds of studies are crucial for the subsequent design of novel hybridization programs and the identification of molecular targets for clone improvement.

## Sensory Profiles and Flavor Chemistry

As mentioned before, CCN 51 is considered by the International Cacao Organization (ICCO) as a bulk cacao (*i.e.,* ordinary cacao) lacking many of the valuable floral, nutty, and fruity attributes identified in premium fine-flavor cultivars. Up to now, two different approaches have been used to determine its sensory profile: (1) a descriptive evaluation by a trained sensory panel (Quintana Fuentes et al., 2015b; Quintana Fuentes, García Jerez & Moreno Martínez, 2018), and (2) metabolic profiling of volatile compounds *via* gas chromatography-mass spectrometry (GC-MS) and GC electronic nose (Kadow et al., 2013; Pallares-Pallares, Perea-Villamil & López-Giraldo, 2016; Rottiers et al., 2019).

Sensory evaluation by trained panels has been performed for CCN 51 liquor obtained from samples collected in different harvesting periods (*i.e.,* 2012, 2013, 2015, and 2018) and from different ecogeographic regions in Colombia by Quintana and co-workers (Quintana Fuentes et al., 2015b; Quintana Fuentes, García Jerez & Moreno Martínez, 2018). The authors defined CCN 51 as a cultivar characterized by strong acidity, bitterness, and astringency with the additional presence of undesirable green flavors. However, their data also revealed the presence of slight notes of fine-flavor attributes (*i.e.,* floral, nutty, and fruity), yet not sufficient to be classified as fine-flavor (Quintana Fuentes et al., 2015b). Interestingly, mixtures of this clone with fine-flavor cultivars have been shown to improve their sensory profile (Quintana Fuentes, García Jerez & Moreno Martínez, 2018) becoming an alternative for flavor enhancement. Nevertheless, research in this regard is still in its infancy and additional experiments still have to be performed to evaluate the sensory profile of popular mixtures used by the farms in different regions.

In 2013, Kadow and Co-workers studied the presence of fine-flavor compounds in CCN 51 by GC/MS and compared their results with the profile of two popular fine-flavor cultivars (Kadow et al., 2013). The authors reported a lower abundance of monoterpenes and secondary alcohols (potential sources of fine-flavor notes) in CCN 51 as compared to the evaluated fine-flavor cultivars. For instance, the presence of monoterpenes like linalool (frequently associated with floral and fruity notes) and other compounds such as isoamylacetate, or undecanone was shown to occur in lower and sometimes undetectable amounts in CCN 51 as compared to fine-flavor cultivars (Kadow et al., 2013). Recently, similar results have been observed when comparing the volatile profile of CCN 51 and four different Nacional cultivars (*i.e.,* EET-103, EET-559, EET-576, and EET-577) (Rottiers et al., 2019). In this recent study, the authors performed a deeper characterization of CCN 51 by using headspace–solid-phase microextraction (HS–SPME) combined with GC–MS (Rottiers et al., 2019). Thus, seventy volatile compounds were identified, grouped in odor/flavor groups and their estimated concentration compared across the evaluated clones as shown in Table 1. GC-MS data confirmed the previously described sensory profile of CCN 51 with a lower content of volatile compounds related to floral and fruity attributes as compared to Nacional cultivars. Interestingly, nutty-associated compounds were found at lower concentrations in CCN 51 as compared to other cultivars or even not detectable as, *e.g.*, is the case for pyrrole-2-carboxaldehyde. As reported in the panel’s evaluation, Rottiers et al. also confirmed the presence of higher concentrations of undesirable compounds (off-flavors) associated with sour, pungent, and rancid attributes in CCN 51 (Rottiers et al., 2019). Interestingly, the concentration of compounds associated with chocolate notes was higher in CCN 51 as compared to the Nacional types, which is also in agreement with the results observed in sensory panel evaluations (Quintana Fuentes et al., 2015b).

The influence of a pre-drying step (before the fermentation process) on the sensory profile of CCN 51 has been determined by HS-SPME-GC-MS (Pallares-Pallares, Perea-Villamil & López-Giraldo, 2016). Drying of the fermentation mass several hours before fermentation was shown to reduce astringency and bitterness (Jimenez et al., 2018b), and resulted in a lower concentration of undesirable compounds (Pallares-Pallares, Perea-Villamil & López-Giraldo, 2016). It is important to mention that some variations in the flavor quality also depend on the conditions under which the fermentation, drying, and roasting processes take place, in addition to the selected cultivar (Luna et al., 2002). Thus, the addition of pre-drying steps, a decrease in the humidity of the bean mass, the assisted increased temperature, and other post-harvest pre-treatments could be alternatives to improve the sensory quality of CCN 51 as it has been shown for other cultivars (Hii et al., 2011). Very recently, a controlled postharvest process for enhancing fine flavor attributes in CCN 51 has been proposed (Santander et al., 2021). The authors developed a laboratory-controlled fermentation using acidic reagents that lead to an improvement in flavor profiling. This kind of study is very promising and further research is required to bring these protocols into large-scale industrial fermentations. In this regard, additional experiments will be required to elucidate a process that can be engineered to enhance fine-flavor attributes in CCN 51 and to reduce the concentration of undesirable compounds.

**Table 1:** Estimated concentration of volatile compounds determined by HSSPMEGCMS of cocoa liquors obtained from beans of CCN 51 and four Nacional type cultivars. (Adapted from Rottiers et al. (2019)). Mean values standard ± deviations are shown.

	Concentration (µg/g liquor)				
Flavor group	CCN 51	EET 103	EET 559	EET 576	EET 577
**Fruity-associated compounds** (e.g., furaneol, 2-Pentanol, Ethyl acetate and Limonene)	4.88 ± 0.12	7.53 ± 0.65	7.20 ± 0.36	6.83 ± 0.43	6.53 ± 0.36
**Floral-associated compounds** (e.g., Linalool, 2-Phenylacetaldehyde, Acetophenone and *β*-Myrcene)	5.80 ± 0.07	6.76 ± 0.06	3.95 ± 0.20	5.22 ± 0.30	5.16 ± 0.26
**Chocolate/nutty-associated compounds** (e.g., Tetramethylpyrazine, 3-Methylbutanal, 2,3-Dimethylpyrazine, and Pyrrole-2- carboxaldehyde)	16.85 ± 0.30	9.54 ± 0.32	12.69 ± 0.76	11.89 ± 0.73	10.30 ± 0.48
**Buttery/creamy-associated compounds** (e.g., 2,3-Butanedione, 2,3-Pentanedione, 3-Hydroxy-2 butanone and Butyrolactone)	4.00 ± 0.15	1.81 ± 0.04	4.75 ± 0.29	2.37 ± 0.11	2.03 ± 0.16
**Undesirable (Off-flavor)-associated compounds** (e.g., Acetic acid, Propionic acid, 3-Methylbutanoic acid, 2-Methylpropanoic acid)	32.92 ± 0.89	23.64 ± 0.77	31.04 ± 2.39	24.31 ± 1.21	25.07 ± 1.97

It is important to mention that up to now, there are no studies related to the identification of bioactive compounds (flavonoids, bioactive peptides, vitamins, etc.) in cacao products obtained from cultivar CCN 51. This is a relevant topic that still must be explored as it could open novel business opportunities for this clone in the nutraceutical, cosmetic and pharmaceutical industry beyond chocolate.

## Physiological Characteristics and Implication for Agronomical Management

### Gas exchange and osmotic adjustment

Cacao is a crop that adapts to shady conditions (Almeida & Valle, 2007), but for CCN 51 it has been found that it has higher CO_2_ assimilation rates (*A*) in open field conditions compared to growing it in shade in agroforestry systems, as reported by Suárez Salazar et al., 2018. For the Amazon region of Colombia, these authors reported maximum rates of *A* of 8 µmol CO_2_ m^−2^s^−1^ at full solar exposure, while cultivation under trees, where the density of photosynthetic photon flux (DFF) that reaches CCN 51 is 400 µmol CO_2_ m^−2^s^−1^ at times of higher radiation, only *A* maxima of 3 µmol CO_2_ m^−2^s^−1^ have been measured. However, in shade, the quantum efficiency is significantly higher (3.8 µmol CO_2_ m^−2^s^−1^), as compared to plants grown at full exposure (3.1 µmol CO_2_ m^−2^s^−1^). The highest rates of *A* in this study at full exposure may be related to the specific leaf area (SLA). In their study, Suárez Salazar et al. reported SLA values of 149 cm^2^ g^−1^ at high radiation and 185 cm^2^ g^−1^ at low radiation (Suárez Salazar et al., 2018). Also, in plants grown under full sun exposure, Jaimez et al. (2018) in Ecuador reported *A* of 8.5 µmol CO_2_ m^−2^s^−1^ and 5.3 µmol CO_2_ m^−2^s^−1^ at DDF of 1000 µmol m^−2^s^−1^ and 400 µmol m^−2^s^−1^, respectively (Jaimez et al., 2018). Under these conditions of full sun exposure, they reported an SLA of 116 cm^2^ g^−1^.

Subsequent trials in CCN 51 seedlings grown under DFF conditions of 2000, 1150 and 630 µmol m^−2^s^−1^ showed significant decreases of *A* (4 µmol CO_2_m^−2^s^−1^) under DFF 2000 µmol m^−2^s^−1^ in comparison to the other two radiation conditions that showed *A* between 7-8 µmol CO_2_ m^−2^s^−1^. Low and significant *A* at high radiation is also manifested in other cultivars (ICS 1, ICS 95, Luker 40, and Luker 50). (Suárez Salazar et al., 2021). Decreases in *A* at high radiation rates are related to also significant decreases in foliar water potentials (Suarez Salazar et al., 2021). However, a characteristic that stands out of CCN 51 is the increase in the non-photochemical quenching of Chla at higher radiation as compared to other cultivars (Suarez Salazar et al., 2021) which allows greater heat dissipation of Chl excitation and protection of the photosynthetic apparatus. CCN 51 has exhibited *A* less than 2.8 µmol CO_2_ m^−2^s^−1^ in plants of 1.5 years of age, planted in pots and growing under conditions of DFF below 400 µmol m^−2^s^−1^ (Baligar et al., 2008; Baligar et al., 2021), which coincides with field values reported by Suárez Salazar et al. (2018) for shaded plants in agroforestry systems (Suárez Salazar et al., 2018). The increase in *A* rates under high radiation conditions in CCN 51 is also reported for other Ecuadorian clones with *A* values similar to those obtained in CCN 51 (Jaimez et al., 2018). It seems that in environments of high cloudiness that predominate at the Ecuadorian coast and in the Colombian Amazon, growing cacao under shady conditions can limit its maximum photosynthetic capacity due to excess shade. In addition, keeping the cacao under excessive shade can increase the incidence of frosty pod rot infection (*Moniliophthora roreri*) (Gidoin et al., 2014).

Under dry conditions without irrigation, Tezara Fernández et al. (2020) reported *A* values of around 5 µmol CO2 m^−2^s^−1^ with DFF of 1000 µmol m^−2^s^−1^ in the province of Esmeraldas, Ecuador. Despite the decrease in *A* rate in the dry period, CCN 51 maintained a higher yield (above 1200 kg ha^−1^). Like other cacao cultivars in Ecuador, CCN 51 maintains higher rates of stomatal conductance (between 250–350 mmol m^−2^ s^−1^) (Tezara Fernández et al., 2020; Jaimez et al., 2018). In these high conductances in plantations under full sun exposure, CCN 51 exhibits water use efficiency (WUE) around 2.3 mmol mol^−1^. Concerning other Ecuadorian cultivars, they are intermediate values (Tezara Fernández et al., 2020; Jaimez et al., 2018), Other cultivars of Criollo type in field conditions have shown greater WUE in dry periods (3.5−4.5 mmol mol^−1^), and in other cultivars, higher WUE has been obtained in periods of rain (Ávila Lovera et al., 2016).

Several cacao cultivars have shown osmotic adjustment (Rada et al., 2005; Almeida et al., 2002), which has been attributed to increases in K and P under conditions of water deficit (Almeida et al., 2002). In cacao, it has previously been shown that maintaining higher osmotic adjustments contributes to a lower number of dead juvenile plants during the dry season (Araque et al., 2012). In the case of CCN 51, using 18-month-old juvenile plants sown in pots and subjected to different amounts of irrigation (varying between 100 to 40% of evapotranspiration), it has been observed osmotic adjustments of 0.60 MPa in plants watered with 60% of evapotranspiration (Arteaga, 2020). Table 2 summarize the osmotic potential at full turgor (Ψ _π100_), at the point of turgor loss (Ψ _π0_) and, the osmotic adjustment obtained for 6 years old trees (from 3 clones (EETP-800, EETP-801 and CCN 51)) in field conditions during the dry and rainy season in Quevedo, Ecuador, according to methodology reported by Tyree & Jarvis (1982) and Schulte & Hinckley (1985). EETP-800 and EETP-801 are cultivars recently released by the INIAP (Loor-Solórzano et al., 2019) and have as common parents the cross between CCN 51 ×EET-233. Interestingly, the latter has shown a low susceptibility to frosty pod rot disease and a medium incidence of witch’s broom infection (*Moniliophthora perniciosa*) (Jaimez et al., 2020). CCN 51 showed the highest osmotic setting of 0.46 MPa, while EETP-800 and EETP 801 showed an osmotic adjustment of 0.40 and 0.43 MPa, respectively.

**Table 2:** Osmotic potentials at saturation (Ψ_*π*_^**100**^), turgor loss point (Ψ_*π*_^**0**^) and osmotic adjustment (OA) of Ecuadorian cocoa cultivars. Data reported by Pichilingue Experimental Station, Ecuador. Mean values of three curves ± standard errors are shown.

Cultivars	Season	Ψ_*π*_^**100**^(MPa)	Ψ_*π*_^**0**^(MPa)	OA (MPa)
EETP-800	Rainy Dry	−1.20 ± 0.08a^*^ −1.60 ± 0.05A	−1.55 ± 0.08a^*^ −2.11 ± 0.18A	0.40
EETP-801	Rainy Dry	1.48 ± 0.09b^*^ −1.71 ± 0.22B	−1.84 ± 0.02b^*^ −2.55 ± 0.02B	0.43
CCN 51	Rainy Dry	−1.14 ± 0.07a^*^ −1.60 ± 0.12A	−1.45 ± 0.10a^*^ −2.24 ± 0.30A	0.46

**Notes.**

Different letters for the same variable indicate significant differences according to the Tukey test (*p* < 0.05). Uppercase letters for the dry season, lowercase for the rainy season.

* Significant differences between seasons for each clone according to Student’s T (*p* < 0.05).

Another important physiological trait that has been recently reported for CCN 51 is its low Cd intake (Argüello et al., 2019; Barraza et al., 2019; Arevalo-Hernandez et al., 2021). For example, an average of 1.4 times higher concentration of Cd has been found in beans of National types when compared to CCN 51 (Argüello et al., 2019). Similar trends have been also reported by Barraza et al. (2019). Remarkably, Arevalo-Hernandez et al. (2021) in an evaluation of 53 genotypes have included CCN 51 between the top 11 genotypes showing a low accumulation of Cd.

Regarding its agronomical management, it seems convenient for CCN 51 in the seedling stage to avoid high radiation due to decreases in *A* and possibly leading to lower growth rates. This aspect must be taken when planning new plantations in regions with high radiation throughout the year. However, the ability of photoprotection mechanisms associated with increased xanthophyll’s cycle exhibited by CCN 51 allows it to avoid drastic damage to the photosynthetic apparatus due to the excess radiation received in the juvenile stages. Already in the productive stage CCN 51 has shown higher *A* at full sun exposure but in conditions where there is low evaporative demand due to the predominance of high cloudiness (Suarez Salazar et al., 2018, Jaimez et al., 2018). It would be appropriate to evaluate the effect of partial shade conditions for at least the first two years on growth and production in regions with high DFF throughout the year. The irrigation and fertilization conditions that occur in large commercial plantations undoubtedly offer less stressful conditions, avoiding important reductions in water potentials that lead to stomatal closures and consequently lower *A* rates. Its osmotic adjustment capacity and its median WUE allow the CCN 51 to tolerate periods of water deficit and probably fewer juvenile plant deaths. For small producers that mostly lack irrigation services, this trait offers a great advantage as compared to other more susceptible cultivars.

### Production dynamics

**Figure 3:**
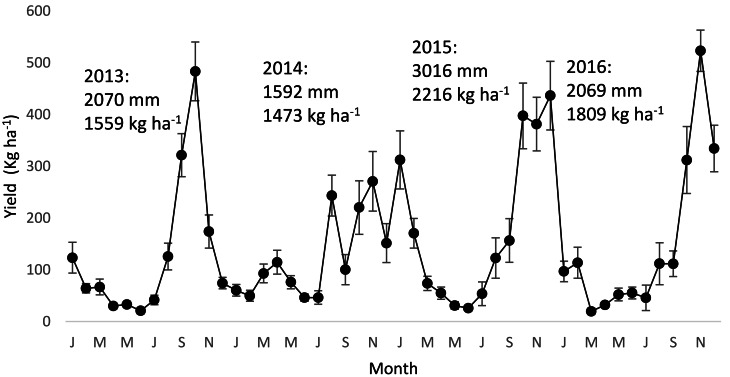
Monthly yield dynamics of CCN 51 in Quevedo province of Los Rios (Ecuador) - Rio Lindo Alto Farm- in the period 2013-2016. Irrigated plantation. Precipitation and total yield for each year. Bars are standard errors of the mean. Each point is the average of eight plots. Historical data provided by the cocoa producing company Agrotropical S.A.

In Ecuador, the highest yields of CCN 51 occur between October - December, regardless of the amount of water supplied to the plantation. For example, in the province of Los Ríos, where the average rainfall is 1800 mm and plants received irrigation, the highest yield in the period 2013-2016 was observed between the months of October-November as shown in Fig. 3. The yield values with irrigation range from 33 to 50 quintals (equivalent to 1500 and 2300 kg ha^−1^, respectively), while the average yields without irrigation reach average values of 1050 kg ha^−1^ in the localities of Quevedo, Tenguel, and Esmeraldas, Ecuador (Sánchez-Mora et al., 2015; Tezara Fernández et al., 2020). This is a high yield considering that these areas are experiencing 5 months without rain. The high-yield Nacional -type clones from the Centro de Cacao Aroma Tenguel-CCAT collection, in the same trial showed yield averages between 200 and 590 kg h^−1^ which is between 80 to 44% lower compared to those obtained with CCN 51 (Sánchez-Mora et al., 2015). In other evaluations with 5-year records for the Quevedo region, CCN 51 showed superior yields between 50 to 70% higher, compared to Nacional-type clones (Boza et al., 2014). In Colombia, average yields between 1000 and 2000 kg ha^−1^ have been registered. It seems that the high variation depends on the fertilization program (Puentes-Páramo, Menjivar-Flores & Aranzazu-Hernández, 2014).

It is known that the amount of precipitation is a factor that notably influences cacao production (Zuidema et al., 2005). However, the higher production at the end of the dry period of CCN 51 in the Ecuadorian coast implies changes in the distribution of assimilate between the vegetative and reproductive parts. Very little is known regarding the distribution of assimilates in cacao (Lahive, Hadley & Daymond, 2018). Therefore, is important to understand why despite water limitations pods grow. This period is also characterized by a low incidence of DFF in this region. It seems the low DFF and the lower vapor pressure deficit (DPV) is a result of high relative humidity (RH) and cloud cover, thus creating a favorable condition of less evaporative demand and less stress, which may favor the continued productivity of CCN 51. It is likely that in the dry period, the growth rate of the pods is lower due to lower rates of *A*, but it seems that a greater amount of assimilates are destined for the growth of pods. The characteristic of the fruit ripening period during the dry season influences a lower incidence of fungal diseases such as frosty pod rot disease (Ploetz, 2016) or pod infections by witch’s broom *M. perniciosa* (Sánchez-Mora et al., 2015). Environmental conditions in the dry period thus increase the growth of fruits in CCN 51. The physiological mechanisms that induce high flowering during the late rainy/early dry period are unknown and it has usually been reported that the largest numbers of flowers occur during the rainy period (Adjaloo, Oduro & Banful, 2012). Besides, a lower amount of rain leads to a lower number of cacao pollinating insects (Arnold et al., 2018) which would influence a lower number of fruits, but this is not what happens with CCN 51. Branco et al. (2018) have classified CCN 51 as a self-compatible and cross-compatible clone. Such characteristics could partially explain its higher production, as it is less dependent on pollinators. Moreover, it has been previously shown that its number of retained flowers is higher as compared to other clones cultivated in other countries after 15 days of self-pollination (Branco et al., 2018).

### Floral biology

The flower of CCN 51 is pinkish, with staminodes and style averaging 6.6 and two mm in length, respectively. The ovary reaches a length of 1.9 mm and has 46.2 ovules (Perea et al., 2017). A significantly greater number of flowers and pollinated flowers have been reported for CCN 51 than for Nacional cacaos that were used in 2010 in Ecuador (Montoya Bazán, 2010). The greater quantity of pollinated flowers can be related to pollen viability as recently found by García Talledo et al. (2019), who have reported that the viability of CCN 51 pollen lasts around 24 h, while in 10 cultivars of the Nacional type, viability is between 3 and 7 h (García Talledo et al., 2019). This gives an advantage for CCN 51 as a possible pollinator. The greater longevity of pollen can influence pollination and produce a high number of seeds compared to other clones. Additionally, CCN 51 has around 44,000 pollen grains per flower, above the average for Nacional cacaos (33,000 grains per flower) (García-Cruzatty et al., 2020) and a higher average number of pollen grains have been found to reach stigmas per flower per month (19.4) than other clones used at the Ecuadorian coast (Mena-Montoya et al., 2020). It has also been recorded that the highest number of grains arriving at the stigmas occurs in February (rainy season) and the lowest number between October and December, *i.e.,* the last months of the dry period (Mena-Montoya et al., 2020). This suggests a reduction in pollen production and flowers due to the lower availability of water that leads to a lower probability of seed production. Also, a lower percentage of falls of small immature pods (cherelles wilt) has been reported in CCN 51 (Montoya Bazán, 2010), which corroborates its advantages for higher production.

CCN 51 has a low pod index (number of pods to get one kilogram of dried cacao- value (15.2)) as compared to other higher National-type cultivars (values between 17.8 and 22.8) (Jaimez et al., 2018). Similar values have been obtained in several locations in Ecuador and Colombia (Quintana Fuentes et al., 2015a; Perea et al., 2017; Jaimez et al., 2018). Its low value is related to an average of 71 seeds in 100 g and an average weight per seed that varies between 1.54 g in Ecuador (Boza et al., 2014) and 1.6 g in Colombia (Quintana Fuentes et al., 2015a). This seed weight is higher than those reported for Nacional-type cacao, suggesting that CCN 51 allocates a larger amount of photoassimilates to the development of seeds, more than for other parts of the pods. Other clones with a higher quantity of seeds in 100 g (between 94 and 85) present averages of weight per seed of 1.2 and 1.17 g (Boza et al., 2014).

## CCN 51 Current Challenges and Perspectives

As discussed in this review, the high production and great adaptability of CCN 51 to various environmental and soil conditions have propelled its expansion into several countries of Latin America. The availability of this clone, its increasing popularity, and its advantages/challenges (Fig. 1) call for some actions that should be done to guarantee its appropriate use by the local farmers.

### Efforts to provide alternative cultivars to local farmers

One current challenge for CCN 51 utilization is to further optimize its agronomical management and to increase its use in breeding programs. The current evidence suggests that the osmotic adjustments observed in CCN 51 are a valuable trait conferring a greater tolerance to water deficit, which might represent an advantage for small farmers without an appropriated irrigation system. Nevertheless, additional research in this field is still required to further evaluate the effect of water supply on yield, as the current evidence suggests the possibility to achieve higher efficiency in the use of water in this clone. The review data suggest that during the end of the rainy season and the first three months of the dry season, pollination occurs preferentially, and consequently in the following dry months the development of pods reaches their highest production rate (October-December period). This occurs independent of irrigation, but with higher production in irrigated plants. The higher production also can be associated with a higher number of pollen grains that ensure better pollination success. Its high seed weight leads to a low value of the pod’s index. Unfortunately, all these physiological characteristics in response to environmental factors have not yet been widely used in breeding programs with goals to obtain cultivars with better adaptation to future climate change scenarios.

Undoubtedly, the development of novel cacao cultivars must be oriented to obtain clones not only with high productivity but also showing a low demand for fertilizers and displaying a high tolerance to diseases, thus reducing the use of fungicides and pesticides. For example, two clones have been recently released in Ecuador by INIAP (EETP 880 and EETP 801) using CCN 51 as parental. These clones have shown higher productions as compared to CCN 51 (Jaimez et al., 2018) and keep both the National-type organoleptic qualities (Loor-Solórzano et al., 2019) and high tolerance to frosty pod rot disease (Jaimez et al., 2020). It is noteworthy that the improvement of cacao is limited by the length of the breeding cycle of the crop that can take at least a decade and these efforts should start soon (Bekele & Phillips-Mora, 2019).

### Efforts to develop an optimal agronomical management

The higher energy dissipation capacity of CCN 51 allows its cultivation under full sunlight exposure. CCN 51 has shown consistent and high yields when growing alone (*i.e.,* monocultures under full sun exposure). Unfortunately, this has led to the displacement of other premium cultivars with excellent flavor potential and other interesting characteristics, thus reducing the diversity in the producers’ farms. For example, Melo & Hollander (2013) point out that between 2002 and 2009, 38% of the interviewed farmers had renewed their plantations using only CCN-51 and sowing it in full sun. On the other hand, the strong increase of this clone in cultivation bears risks of monoculture in cacao such as a higher incidence of pests and diseases (Andres et al., 2016). Diversification will reduce dependence on a single cultivar prone to the risk of being eliminated by the high incidence of insects and diseases (Niether et al., 2020). Therefore, avoiding monoculture protects against devastating losses and would also increase the likelihood of a recovery in less time. Probably each country should implement the possibility of cultivating CCN 51 combined with other cultivars that have been successfully used in each region. Unfortunately, the co-cultivation of CCN 51 and premium clones has resulted in the incorrect tendency to mix beans of this clone with premium cacao beans aimed to increase farm productivity. This has been shown to directly affect the sensorial quality of the final product (*i.e.,* premium chocolate) with deleterious economic consequences. In this regard, as previously discussed, several methodologies have been developed to assure traceability based on the identification of selected molecular markers for each clone. Nevertheless, further research still must be done in this regard to discouraging these bad agronomical practices. Monocultures of CCN 51 (*i.e.,* without shade trees or in the presence of temporary shade species) can lead to a faster depletion of the soil (Andres et al., 2016) and loss of pollinators, which significantly influences the production (Groeneveld et al., 2010). For this reason, despite its good production under full sunlight exposure, different production models (*i.e.,* agroecological farming systems) that combine CCN 51, and shade trees still must be designed, implemented, and transferred to the local farmers.

An important advantage of the cultivation of CCN 51, is its lower accumulation of Cd as compared to other clones. This is certainly a field that still must be explored as Cd uptake is a current bottleneck to access the European premium markets.

### Efforts to develop an optimal post-harvest management

Generally, CCN 51 is considered bulk cacao with negative impacts in the fine-flavor market. However, as mentioned before, several alternatives have been proposed to further engineer the post-harvesting process to demystify its utilization as a potential clone for controlled production of fine-flavor cacao either by standardized controlled fermentations, or by breeding, or both, to eventually enhance its sensory profile to unite the best of the two worlds. Research in this topic is still in its infancy and additional experiments will be required to engineer a process that redefines the flavor profile of CCN51, thus opening better opportunities for this clone in the complex cacao market.

## Additional Information and Declarations

### Data Availability

The following information was supplied regarding data availability: This is a literature review.
